# Decadal Trends and Common Dynamics of the Bio-Optical and Thermal Characteristics of the African Great Lakes

**DOI:** 10.1371/journal.pone.0093656

**Published:** 2014-04-03

**Authors:** Steven Loiselle, Andrés Cózar, Enyew Adgo, Thomas Ballatore, Geoffrey Chavula, Jean Pierre Descy, David M. Harper, Frank Kansiime, Ismael Kimirei, Victor Langenberg, Ronghua Ma, Hugo Sarmento, Eric Odada

**Affiliations:** 1 Dipartimento di Biotecnologie, Chimica e Farmacia, Consorzio per lo Sviluppo dei Sistemi a Grande Interfase, Università degli Studi di Siena, Siena, Italy; 2 Departamento de Biología, Universidad de Cádiz, Puerto Real, Spain; 3 College of Agriculture and Environmental Science, Bahir Dar University, Bahir Dar, Ethiopia; 4 Lake Basin Action Network, Moriyama, Japan; 5 Department of Civil Engineering, University of Malawi - The Polytechnic, Blantrye, Malawi; 6 Department of Biology, University of Namur, Namur, Belgium; 7 Department of Biology, University of Leicester, Leicester, United Kingdom; 8 Department of Environmental Management, Makerere University, Kampala, Uganda; 9 Kigoma Research Centre, Tanzanian Fisheries Research Institute, Kigoma, Tanzania; 10 DELTARES, Delft, The Netherlands; 11 Nanjing Institute of Geography and Limnology, Chinese Academy of Sciences, Nanjing, China; 12 Federal University of Sao Carlos, Department of Hydrobiology, São Carlos, Brazil; 13 Geology Department, University of Nairobi, Nairobi, Kenya; University of Vigo, Spain

## Abstract

The Great Lakes of East Africa are among the world’s most important freshwater ecosystems. Despite their importance in providing vital resources and ecosystem services, the impact of regional and global environmental drivers on this lacustrine system remains only partially understood. We make a systematic comparison of the dynamics of the bio-optical and thermal properties of thirteen of the largest African lakes between 2002 and 2011. Lake surface temperatures had a positive trend in all Great Lakes outside the latitude of 0° to 8° south, while the dynamics of those lakes within this latitude range were highly sensitive to global inter-annual climate drivers (i.e. El Niño Southern Oscillation). Lake surface temperature dynamics in nearly all lakes were found to be sensitive to the latitudinal position of the Inter Tropical Convergence Zone. Phytoplankton dynamics varied considerably between lakes, with increasing and decreasing trends. Intra-lake differences in both surface temperature and phytoplankton dynamics occurred for many of the larger lakes. This inter-comparison of bio-optical and thermal dynamics provides new insights into the response of these ecosystems to global and regional drivers.

## Introduction

**Figure 1:**
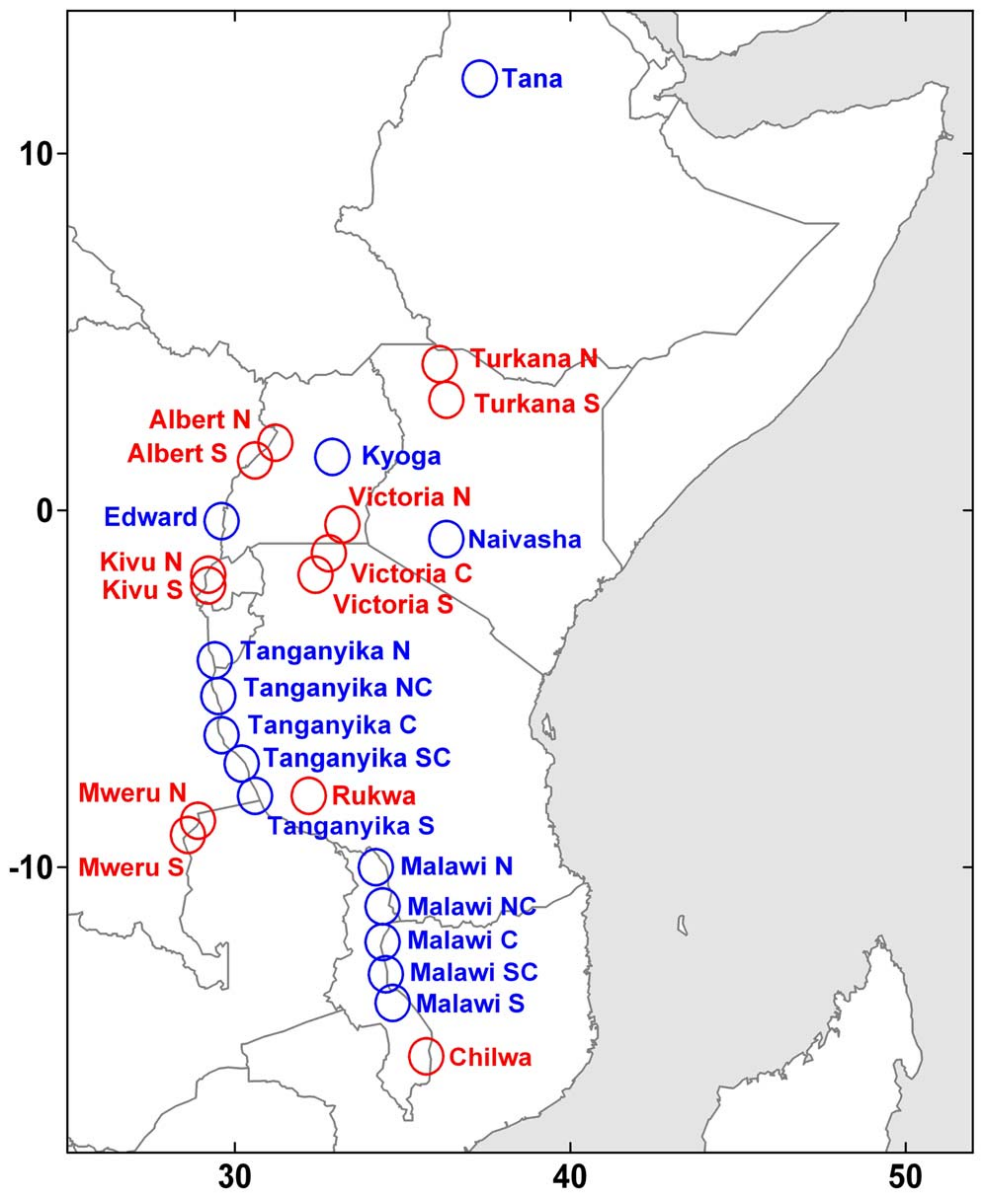
Lakes and lake sections included in the present analysis of the African Great Lakes.

The Great Lakes of East Africa are among the world’s most important aquatic ecosystems from the point of view of ecosystem services, biodiversity and carbon cycling [1]. They are highly heterogeneous, presenting an array of hydrological and biological characteristics, ranging from shallow eutrophic water bodies (e.g. Lake Victoria) to deep oligotrophic lakes (e.g. Lake Tanganyika). Recent studies have demonstrated linkages between climate variability and productivity of several African lakes [2], [3], [4]. This is partially due to the importance of direct rainfall and evaporation in the water balances in the larger lakes (e.g. Lakes Tanganyika, Malawi and Victoria) and river flow in the smaller lakes (e.g. Lake Turkana) as well as climate driven lake stratification cycles [5], [6], [7]. Inter-annual variations in regional and global climate patterns have a strong influence on monsoon dynamics, in particular the Inter Tropical Convergence Zone (ITCZ) [8]. Local and regional impacts also play an important role on the chemical and biological dynamics of many of these lakes [9], [10], [11]. Remote measurements have been used to explore the spatio-temporal variability of the temperature and bio-optical properties in several of these lakes [12], [13], [14]. However, a systematic inter-lake comparison using a common methodology has yet to be performed. There are multiple reasons for this, geographical (e.g. altitude) and seasonal differences in atmospheric optical conditions and seasonal differences in aquatic optical properties. Most importantly, there are limited data for lake specific algorithm development and validation while standard bio-optical algorithms have been predominately developed for phytoplankton dominated water bodies found at sea level [15]. In the present study, we make a comparative analysis of the thermal and bio-optical dynamics of thirteen lakes lying in or between the two branches of the Great Rift in Eastern-Central Africa; Lakes Albert, Chilwa, Edward, Kivu, Kyoga, Malawi, Mweru, Naivasha, Rukwa, Tana, Tanganyika, Turkana and Victoria (Figure 1). Using site-specific seasonal smoothing of the time series, we examine their decadal trends. These trends are also compared to interannual climate drivers, allowing us to explore commonalities between lakes and potential drivers of change.

## Materials and Methods

Monthly estimates of chlorophyll-a concentrations and lake surface temperatures (night) were obtained as Level 3 data from the MODIS AQUA mission dataset from the Goddard Earth Sciences Data and Information Services Centre (http://disc.sci.gsfc.nasa.gov/giovanni, Ocean Color Radiometry Online Visualization and Analysis Monthly Data), following Reprocessing 1.1 [16] and covering the period from July 2002 to July 2011.

Lake sections (64 km^2^–128 km^2^) were delineated using MODIS AQUA data at 4 km spatial resolution for the bio-optical estimates (MAMO_CHLO_4 km). Surface temperature estimates of the same lake section were obtained using MODIS AQUA data at 9 km spatial resolution (MAMO_NSST_9 km). Larger lakes were divided into separate lake sections based on the large scale patterns identified in previous studies or where lake dimension suggested such patterns could be possible [17], [14]. Possible influences of coastal waters and edge effects were minimized in the large lakes by selecting lake sections at least 10 km from the lake border.

To avoid errors due to geographical differences between the optical conditions (atmospheric and aquatic) of the African lakes and those of standard MODIS processing, each record category was converted to a vector of anomalies by subtracting the lake wide average and dividing by the lake wide standard deviation. In such a manner, consistent spatial differences in optical conditions between lake sections would not influence the analysis of lake dynamics [17].

To remove the influence of seasonal variability in optical conditions (atmospheric and aquatic) between lakes and lake sections, each vector underwent a seasonal smoothing by using a yearly centred moving average with a length of 12 (months). The resulting trend vector for each parameter was then subtracted from the original data and the differences were averaged across all contemporary (monthly) observations to produce a seasonal index. The trend vector and seasonal indices were subtracted from the data to give a residual component which was checked for normal distribution.

The resulting trend vectors (SST for the trend vector of lake surface temperature anomalies, CHLa for the trend vector of chlorophyll a anomalies) were independent of seasonal and intra-lake spatial variations in optical conditions (atmospheric and aquatic). These vectors of seasonally smoothed data anomalies were then used to compare dynamics between lakes and lake sections to determine commonalities in inter-annual trends.

Seasonally smoothed trend vectors of meridional wind speed anomalies (WIND) and precipitation rate anomalies (RAIN) for each lake section were constructed using NCEP/NCAR data [18], [19] (http://www.esrl.noaa.gov/psd/data/gridded/data.ncep.reanalysis.html). Likewise, a trend vector of the Multivariate El-Niño Southern Oscillation Index (MEI) was also constructed [20] (http://www.esrl.noaa.gov/psd/enso/mei/). To examine the role of the ITCZ, we used historical dekadal (10-day) data for the African position of the ITCZ from April 2003 to July 2011 provided by the NOAA CPC Africa Desk/International Desk's Team (www.cpc.ncep.noaa.gov/products/fews/ITCZ/itcz) [21]. Monthly averages of dekadal positional data for longitudes of 30 and 35 were averaged and compared to SST and CHLa anomalies.

Factor analysis was utilised to identify common temporal co-variation between lake sections. This analysis was based on a linear combination of variables which contained the maximum variance. The reduced numbers of factors (eigenfunctions) is orthogonal (uncorrelated) while the eigenvalue of each factor was used to determine the fraction of the variability explained. In this manner, the temporal dynamics of the 27 lake sections was reduced to a smaller number of dominant factors. Common temporal modes were identified by determining the correlation between each lake section and the dominant factors for each variable. Lake sections with similar temporal dynamics were correlated to the same factor. It should be noted that this approach assumes that the temporal variability of each variable dataset is due to common forcing agents.

Decadal trends and correlations between drivers and anomaly vectors were determined by using linear model with a monthly time scale where direct correlations (increasing) had positive Pearson correlation coefficients (r) and indirect correlations (decreasing) had negative coefficients.

## Results

The SST trend vectors of each lake section (27), analyzed by factor analysis, had five significant temporal modes which accounted for 92.5% of the total variability of the dataset (60.2%, 14.0%, 9.0%, 5.6%, 3.8% respectively). Sections of the same lake most often followed the same temporal mode (Table 1).

**Table 1:** Dominant factors, decadal (2002–2011) trends and correlations between bio-optical, thermal and climate trends in 27 lakes sections of the African Great Lakes (n = 100 for SST, CHLa and MEI, n = 60 for ITCZ).

Lake section	Dominant factors				Trends (r)		Correlation between parameters (r)					
	CHLa	SST	WIND	RAIN	CHLa	SST	CHLa MEI	SST MEI	CHLa SST	SST ITCZ	CHLa ITCZ	
L. Tana		2	3	4		**−0.61**		0.03		0.08		
L. Turkana north	3	3	2	1	**−0.61**	0.25	**0.54**	0.00	0.28	**−**0.27	**−**0.22	
L. Turkana south	3	3	2	1	**−0.53**	0.27	**0.56**	**−**0.05	0.18	**−0.41**	**−**0.11	
L. Albert north	1	2	3	4	**0.67**	**0.58**	**−0.55**	0.11	0.23	**−0.73**	0.06	
L. Albert south	1	2	3	4	**0.61**	**0.49**	**−**0.22	0.19	0.13	**−0.78**	0.10	
L. Kyoga	4	4	3	4	**−0.44**	**0.56**	0.00	**−**0.24	**−**0.08	**−0.75**	0.07	
L. Edward	5	4	3	4	**0.61**	**0.35**	0.03	**−**0.06	**0.40**	**−0.71**	0.21	
L. Naivasha		1	1	1		0.25		0.22		**−0.64**		
L. Victoria north	4	1	1	1	0.11	**−**0.02	**−**0.06	**0.39**	**−**0.28	**−0.80**	**0.58**	
L. Victoria centre	3	1	1	1	**0.35**	**−**0.17	**−**0.20	**0.31**	0.08	**−0.59**	**0.56**	
L. Victoria south	2	1	1	1	0.22	**−**0.17	**−**0.03	**0.33**	0.16	**−0.73**	**0.35**	
L. Kivu north	5	1	1	3	0.09	0.05	**−**0.03	0.29	**−**0.17	**−0.73**	**0.46**	
L. Kivu south	5	1	1	3	**−**0.11	**−**0.11	**−**0.19	0.26	**−0.32**	**−0.66**	**0.37**	
L. Tanganyika north	5	1	1	3	**−0.73**	**−**0.05	**0.36**	**0.44**	**−**0.12	**−0.75**	**0.44**	
L. Tanganyika northcentre	5	1	1	3	**−0.37**	**−**0.06	0.06	**0.31**	**−**0.11	**−0.77**	0.27	
L. Tanganyika centre	4	1	1	3	0.02	0.18	0.07	0.28	**−0.38**	**−0.85**	**0.33**	
L. Tanganyika southcentre	4	1	1	3	0.05	0.29	**−0.35**	0.19	**−0.51**	**−0.88**	**0.41**	
L. Tanganyika south	4	2	1	3	0.13	**0.42**	**−**0.16	0.10	**−0.48**	**−0.86**	**0.37**	
L. Rukwa		4	2	2		**0.47**		0.01		**−0.90**		
L. Mweru north	1	2	2	4	**0.72**	**0.45**	**−**0.10	0.12	0.28	**−0.84**	**0.51**	
L. Mweru south	1	2	2	4	**0.62**	0.26	0.03	0.28	0.20	**−0.82**	**0.59**	
L. Malawi north	2	2	2	2	**0.37**	**0.45**	**−**0.12	0.11	**0.37**	**−0.93**	0.00	
L. Malawi northcentre	2	2	2	2	**0.33**	**0.53**	**−**0.10	0.02	**0.42**	**−0.93**	0.19	
L. Malawi centre	2	2	2	2	0.4	**0.55**	**−**0.10	**−**0.01	0.25	**−0.93**	**0.39**	
L. Malawi southcentre	2	2	2	2	**0.58**	**0.56**	**−**0.09	**−**0.19	0.16	**−0.92**	0.30	
L. Malawi south	1	2	2	2	**0.61**	**0.67**	**−**0.20	**−**0.12	0.24	**−0.89**	0.15	
L. Chilwa		5	2	2		**0.40**		**−0.31**		**−0.78**		

Significant correlations (p<0.01) are presented in bold.

The SST trend vector for each lake section from 2002–2011, examined using a linear model and decadal trends, was identified as either positive (increasing), negative (decreasing) or not significant at p>0.01 (Table 1, Figure S1 in File S1). The decadal trend for the SST vector was positive for Lakes Albert, Chilwa, Edward, Kyoga, Malawi (all sections), Mweru (both sections), Rukwa, Tanganyika (south and southcentre), and Turkana (both sections), indicating an overall warming of the lake surface waters. A negative trend was observed for Lake Tana. The highest inter-annual variability (lowest r) was found in those lakes south of the equator (Lakes Victoria, Kivu, Tanganyika (north)).

Using the CHLa trend vectors, factor analysis identified five significant temporal modes that accounted for 80.8% of the variability of the dataset (27.0%, 24.8%, 13.5%, 8.6%, and 6.9%, respectively). Differences in the inter-annual trend between sections of the same lake were evident in Lakes Tanganyika and Victoria. Positive trends (p<0.01) in CHLa occurred in Lakes Albert, Edward, Malawi (all sections), Mweru (both sections) and Victoria (centre) (Table 1, Figure S1 in File S1). Negative trends were evident in Lakes Kyoga, Tanganyika (north and north centre) and Turkana. The highest inter-annual variability was in the smaller lakes as well as the south section of Lakes Tanganyika and Malawi.

Factor analysis of WIND and RAIN identified three and four dominant modes that accounted for 89.9% and 95.2% of the variability of each dataset. Negative trends in WIND were observed in most lakes at or above the equator, while positive trends occurred in lakes below the equator. Positive trends in RAIN were observed in all lakes except Lakes Malawi and Mweru (negative) and Lake Tanganyika (not significant).

The multivariate ENSO index (MEI), used to compare inter-annual global climate variations with the SST and CHLa dynamics in each lake section (Table 1, Figure S2 in File S1), showed a significant positive correlation between MEI and SST (p<0.01) in sections of Lakes Victoria, Tanganyika, Kivu and Mweru, while a negative correlation for Lake Chilwa. Correlations between CHLa trends and MEI were both positive and negative (Table 1) with intra-lake differences. The decadal trend of MEI during the study period was negative and included the 2007–2009 and 2010–2011 La Niña events and the 2009–2010 El Niño events. WIND was positively correlated to MEI in most lakes while RAIN was negatively correlated.

The comparison of SST anomalies to ITCZ positional data (Table 1) showed a significant negative correlation between ITCZ and SST (p<0.01) in all lake sections with the exception of Lakes Tana and Turkana (north). The correlation increased at lakes with latitudes below the equator. Correlations between CHLa anomalies and ITCZ positional data were positive in several lakes, most significantly in Lakes Victoria and Mweru (Figure S2 in File S1).

## Discussion

The decadal warming observed in both large and small lakes results from inter-annual variability in radiative balances and latent heat exchange, as well as indirectly from changes in lake mixing and lake water balances. Interestingly, significant warming trends were limited to those lakes which displayed the lowest correlation with MEI. SST trends in Lakes Victoria, Tanganyika (north, north centre and centre) and Kivu (both sections) showed a clear sensitivity to MEI, which led to higher inter-annual fluctuations but no significant decadal trend. The spatial pattern of the MEI-SST relationship confirms that the lakes between 0° to 8° south are most sensitive to El-Niño effect on the ITCZ and the related weakening of the monsoon wind flow over East Africa [23]. Our observations suggest that the effect of El-Niño phenomenon on surface heat balances can be recurrent and significant for many lakes in Tropical East Africa [14]. The high correlation between the SST dynamics and the latitudinal movement of the ITCZ (Table 1) confirms the influence of climate drivers on the dynamics of the African lakes [21], [22].

Trends in CHLa are influenced by inter-annual variability in nutrient availability and optical conditions related to light limitation. Both factors are related to long term trends in local and regional conditions, as well as climate. The Lake Albert CHLa trend had a high negative correlation with MEI while Lake Turkana CHLa (north and south) was positively correlated. Opposing correlations between CHLa trends and MEI in the north and south of Lake Tanganyika were found. The influence of the latitudinal movement of the ITCZ was evident in several lakes (Lakes Kivu, Mweru and Victoria) and individual sections of Lakes Malawi and Tanganyika.

Decadal trends in CHLa in Lake Tanganyika also showed latitudinal differences, while all sections of Lake Malawi demonstrated a positive trend in CHLa over the study period, indicating a general increase in phytoplankton biomass. The centre section of Lake Victoria showed a significant positive trend, while the south section showed a similarly positive but less significant (p = 0.025) decadal trend.

CHLa dynamics are influenced by lake hydrodynamics through variations in seasonal cycles in mixing depth and nutrient upwelling. While SST dynamics are based on thermal emission from surface waters, surface temperature anomalies have been used to track changes in lake hydrodynamics [12], [14]. Lake Tanganyika CHLa (south, southcentre and centre) was negatively correlated to SST. Such a negative relationship would indicate that upwelling nutrient rich waters have a positive impact on phytoplankton productivity [12]. A similar negative relationship was found for the south section of Lakes Kivu. Lake Victoria behaved in an opposite manner, as CHLa and SST were negatively correlated in the north section only, with a weak, but positive relationship in the south. A positive relationship between CHLa and SST could indicate a positive effect of lake stratification on phytoplankton growth (e.g. due to light limited growth). The CHLa trend in the north and centre sections of Lake Malawi showed a positive correlation to SST, as did correlations between SST and CHLa in Lakes Edward, Mweru (north) and Turkana (north).

### Intra-lake Variability in Trend Vectors

In the present dataset, intra-lake differences in trend vectors of SST (Table 1) were significant in Lakes Malawi, Tanganyika, Victoria and Albert (p<0.001) and to a lesser degree in Lakes Mweru (p = 0.01). In Lakes Victoria, Malawi and Mweru, the northern SST trend vectors were continuously higher than the southern lake sections, indicating a stable latitudinal SST difference in each lake. Lake Albert SST trend vectors were highest in the south section. It should be noted that seasonal latitudinal switches in SST may also be present but could remain undetected using anomaly trend vectors. Large scale latitudinal differences in surface temperature have been associated to differences in the exposure to the monsoon winds and the duration of the windy season (e.g. Lake Victoria [14]). On the other hand, the highest SST trend vectors in Lake Tanganyika were found in the north and south sections while the lowest SST vector occurred in the centre section. Large scale latitudinal circulation has been reported in Lake Tanganyika [24], [25].

**Figure 2:**
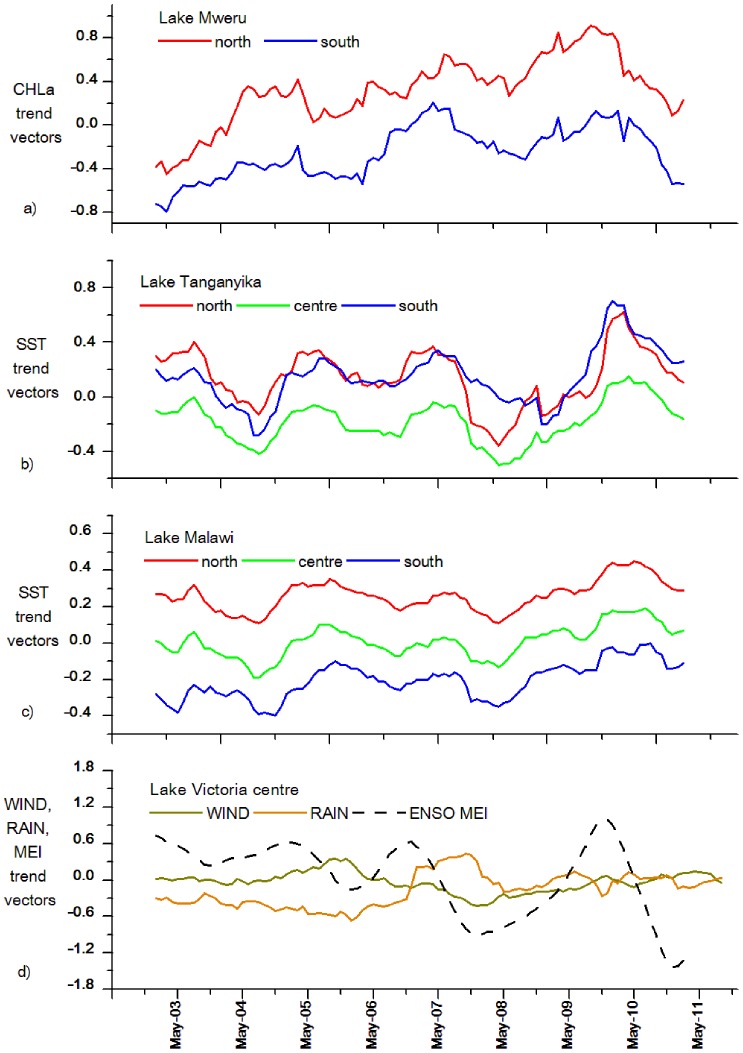
Trend vectors of A) CHLa for north and south sections of Lake Mweru, B) SST for north, centre and south sections of Lake Tanganyika, C) SST for north, centre and south sections of Lake Malawi, and D) WIND and RAIN for Lake Victoria centre section and ENSO MEI.

Intra-lake differences in CHLa trends were significant in most lakes. In Lakes Tanganyika and Malawi, the CHLa trend vectors in the south were significantly higher than those of the northern sections. In Lake Tanganyika, latitudinal differences in chlorophyll a concentration have been confirmed in several studies [26], [27], [28], [29]. Similarly, the south sections of Lake Malawi had been reported to have the highest productivity for most of the year [30], [31]. In Lakes Albert and Mweru (Figure 2), the north section CHLa trend vectors were consistently higher than those of the south. Lake Kivu’s CHLa dynamics showed less clear latitudinal differences, even though differences in phytoplankton composition have been reported, with cyanobacteria more abundant in the north section while diatoms and cryptophytes being a higher proportion of total phytoplankton biomass in the south [32].

## Conclusions

The anomaly trend vectors of SST indicated that a decadal warming has occurred in all of the Great Lakes outside the latitude range of 0° to 8° south. Those lakes unaffected by the decadal warming trend were found to be more sensitive to inter-annual climate fluctuations related to the El Niño phenomena. It should be noted that the warming trends are limited to the study period, but the increased sensitivity of lakes in the latitudinal band of 0°–8° south to El Niño was clear. On the other hand, the sensitivity to the latitudinal position of the ITCZ was elevated in nearly all lakes south of the equator.

The relative response of CHLa to changes in SST, MEI and ITCZ varied between lakes and between sections of the same lake. There was no clear regional trend towards eutrophication, as some lakes showed a negative decadal trend in CHLa, including sections of Lakes Tanganyika, Kyoga and Turkana. Lake Malawi presents a positive decadal trend in CHLa and a positive relationship between SST and CHLa, indicating that this lake is moving to a higher trophic status, with a marked sensitivity to hydrodynamics.

The regional and seasonal differences in the atmospheric and aquatic optical properties of the East African Great lakes, combined with limited field data have hampered a regional analysis. The present approach does not replace the need for extensive field data or for the development of lake specific algorithms to estimate aquatic and atmospheric parameters. However, the use of anomaly trend vectors allows for the identification of common lake trends and possible links between potential drivers and lake responses.

## Supporting Information

File S1 Figure S1. Decadal trends (2002–2011) in anomaly vectors of A) SST and B) CHLa in the African Great Lakes, blue circles for a positive trends, red circles for a negative trends, open circles for no significant trend (p>0.01). Figure S2. Correlations between trend vectors of A) CHLa and MEI, B) SST and MEI and C) CHLa and ITCZ in the African Great Lakes, blue circles for a positive correlations, red circles for a negative correlations, open circles for no significant correlations (p>0.01).
(DOCX)
