# Supplementary figures and images for: Decadal Trends and Common Dynamics of the Bio-Optical and Thermal Characteristics of the African Great Lakes
Source: PLoS One. 2014 Apr 3;9(4):e93656. doi: 10.1371/journal.pone.0093656 (PMC3974808; doi:10.1371/journal.pone.0093656)

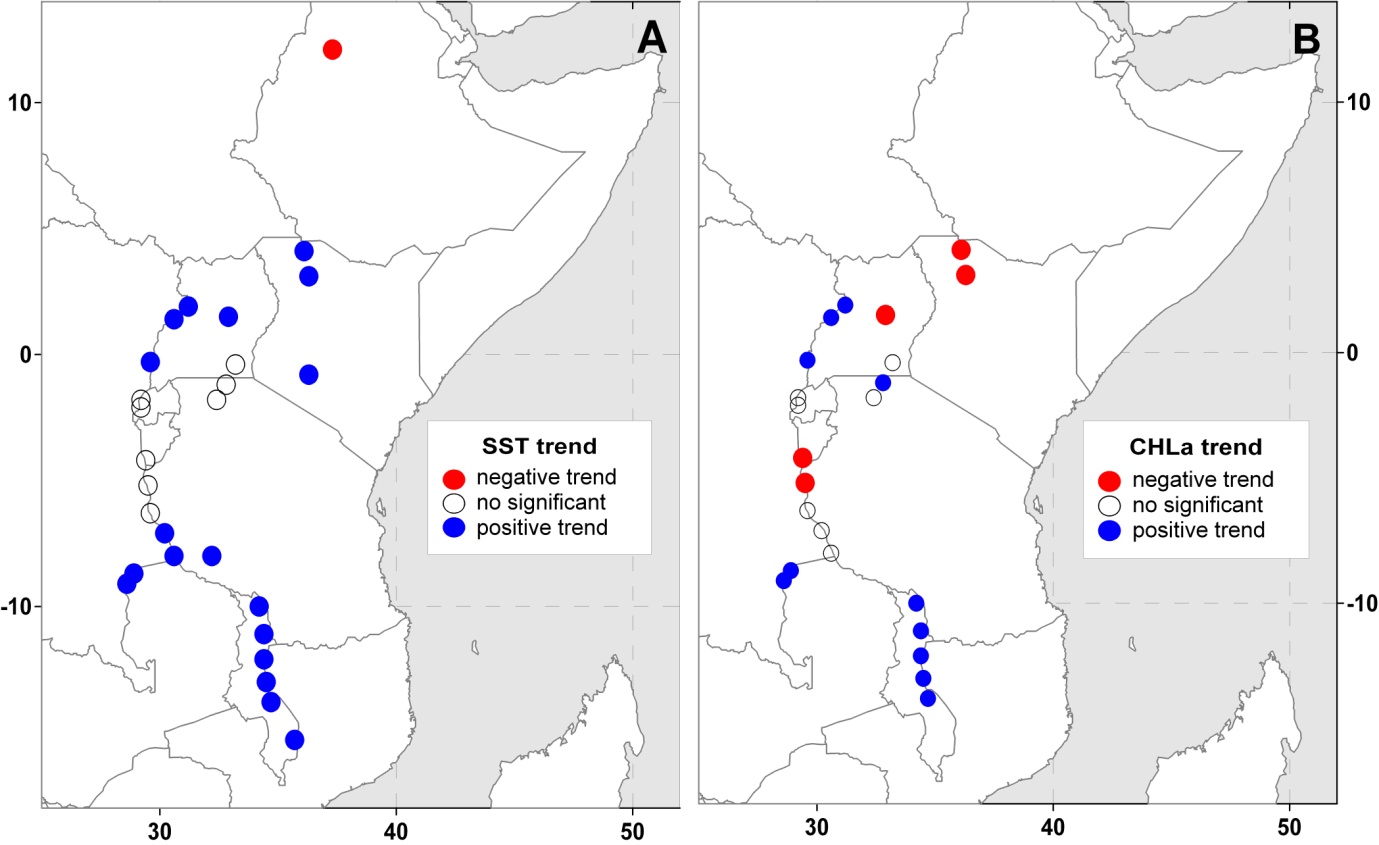


Figure S1.


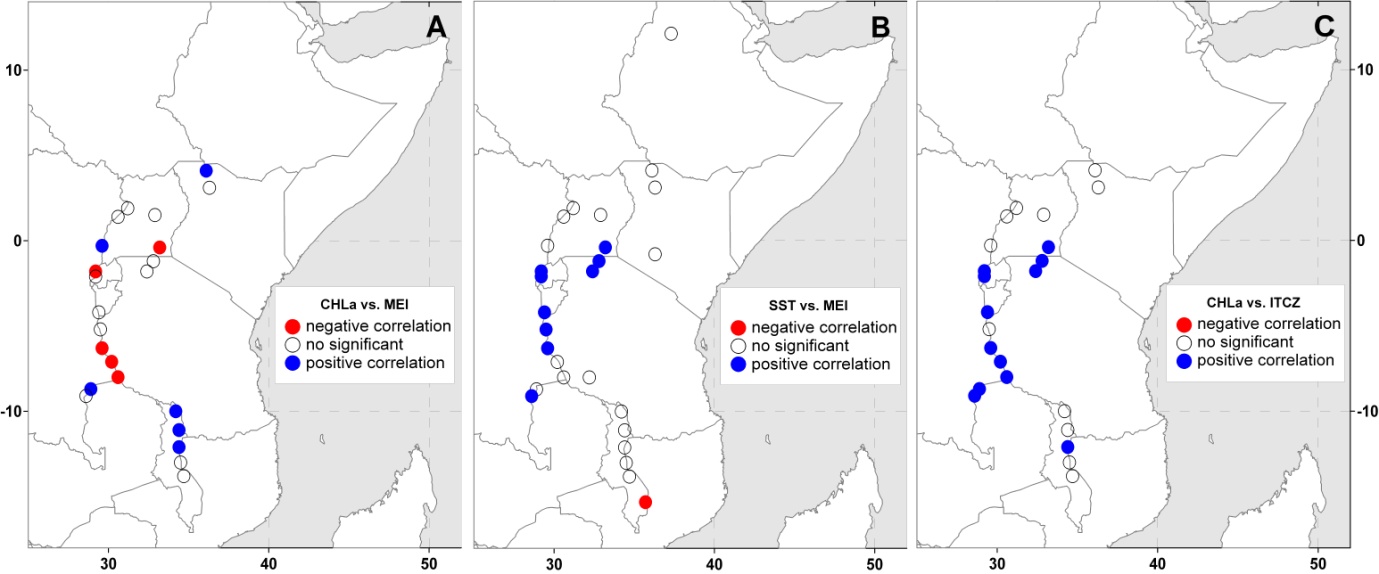


Figure S2

Supplement: File S1 — Figure S1. Decadal trends (2002–2011) in anomaly vectors of A) SST and B) CHLa in the African Great Lakes, blue circles for a positive trends, red circles for a negative trends, open circles for no significant trend (p>0.01). Figure S2. Correlations between trend vectors of A) CHLa and MEI, B) SST and MEI and C) CHLa and ITCZ in the African Great Lakes, blue circles for a positive correlations, red circles for a negative correlations, open circles for no significant correlations (p>0.01). (DOCX) [file pone.0093656.s001.docx]
